# Lateral Duodenal Diverticulitis Mimicking a Right Retroperitoneal Collection: A Case Report

**DOI:** 10.7759/cureus.101578

**Published:** 2026-01-15

**Authors:** Mahmoud Raheem Heidous, Devendra Kumar, Ahmed Hadi, Soumya Hareendranath, Ameer Cheikh Alnajjarin

**Affiliations:** 1 Clinical Imaging Department, Al Wakra Hospital, Hamad Medical Corporation, Al Wakrah, QAT

**Keywords:** abdominal ultrasound, giant duodenal diverticulum, multimodality imaging, retroperitoneal collection, ultrasound diagnosis

## Abstract

Giant duodenal diverticula are rare and often asymptomatic but may present with atypical clinical and imaging findings that resemble intra-abdominal or retroperitoneal collections, abscesses, or cystic neoplasms. We report the case of a 67-year-old male who presented with recurrent right-sided abdominal symptoms initially treated as pyelonephritis and subsequently diagnosed as a right retroperitoneal abscess. Further evaluation with multimodal imaging, including contrast-enhanced computed tomography (CECT) with oral contrast and retrospective ultrasound review, revealed a giant duodenal diverticulum communicating with the second part of the duodenum. Accurate interpretation of these findings allowed revision of the diagnosis and prevented unnecessary percutaneous drainage, thereby avoiding potential iatrogenic complications. The patient was managed conservatively with intravenous antibiotics and demonstrated clinical improvement, with endoscopic evaluation planned for further assessment. This case highlights the value of careful multimodal imaging correlation in avoiding inappropriate invasive procedures and informing optimal patient management.

## Introduction

Duodenal diverticula are the second most frequent gastrointestinal diverticula after diverticula of the colon, with an estimated prevalence of 5-22% on radiologic or endoscopic studies [1,2]. Most duodenal diverticula are asymptomatic and discovered incidentally; however, a small proportion become clinically significant due to inflammation, perforation, hemorrhage, or compression of adjacent structures [3,4].

Giant duodenal diverticula (larger than 4 cm) are uncommon but may mimic abscesses, cystic neoplasms, or retroperitoneal collections on imaging, with lateral duodenal diverticula particularly prone to misdiagnosis due to their mass-like appearance and nonspecific inflammatory changes on CT [3]. They are usually located near the Ampulla of Vater but can occur throughout the duodenum, most often on the medial side [1]. Their proximity to vital retroperitoneal organs, particularly the pancreas and right kidney, often leads to diagnostic confusion and potentially unnecessary invasive interventions such as drainage or surgery [3,5].

This case report presents a patient with a giant duodenal diverticulum initially misdiagnosed as a right retroperitoneal abscess. The report highlights the importance of multimodal imaging correlation and careful interpretation to avoid iatrogenic complications.

## Case presentation

A 67-year-old male with a history of type 2 diabetes mellitus presented to the emergency department with right flank pain, fever, and malaise. Physical examination revealed tenderness in the right flank. Laboratory investigations showed elevated white blood cell count and inflammatory markers, consistent with infection.

**Figure 1:**
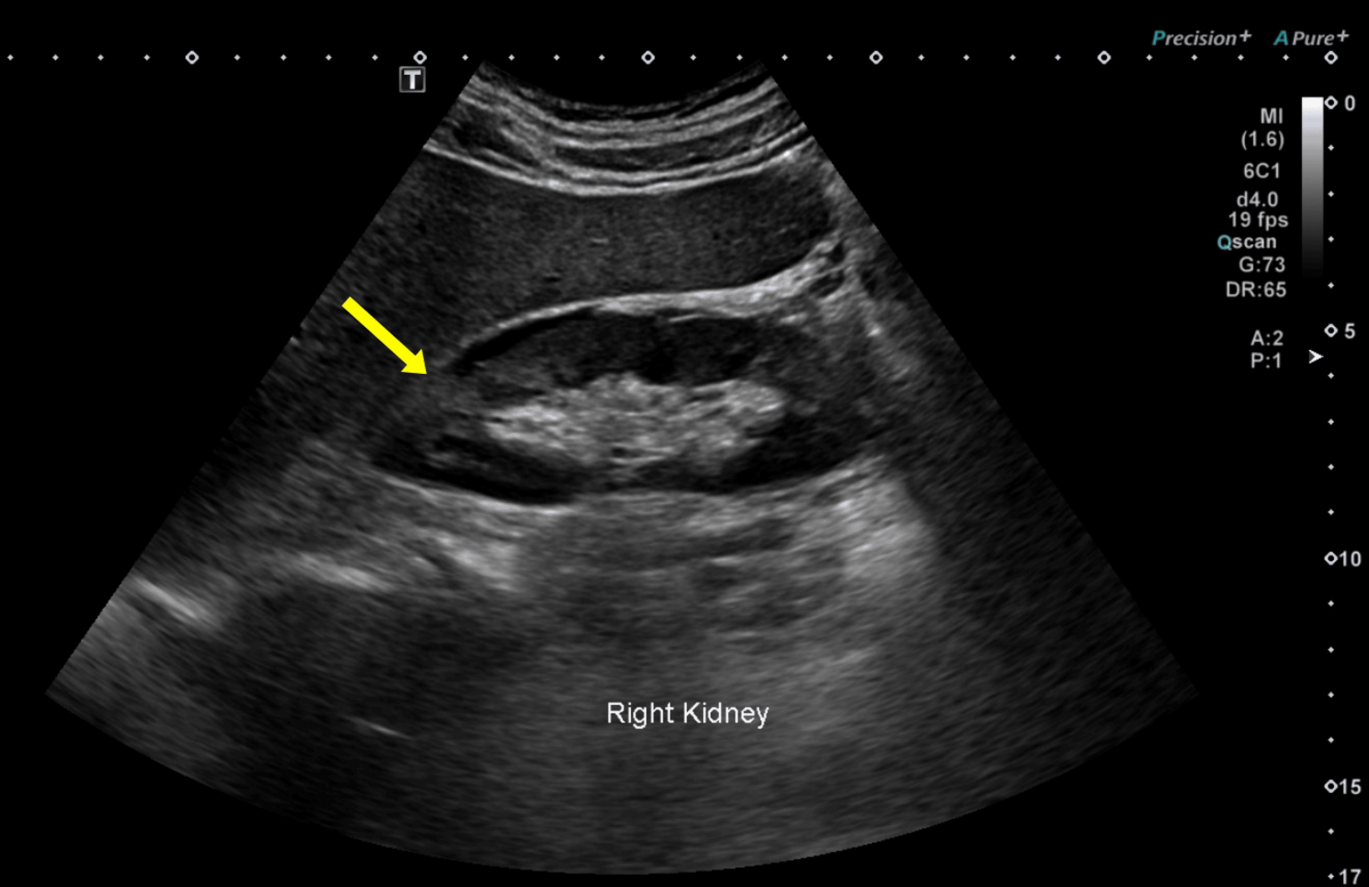
Initial ultrasound showing a hyperechoic area at the upper pole of the right kidney (yellow arrow) with mild perinephric fluid, suggestive of acute pyelonephritis.

An abdominal ultrasound was performed, revealing a hyperechoic, heterogeneous area in the upper pole of the right kidney with mild surrounding fluid, findings suggestive of pyelonephritis (Figure 1). The patient was started on broad-spectrum antibiotics, with clinical improvement.

**Figure 2:**
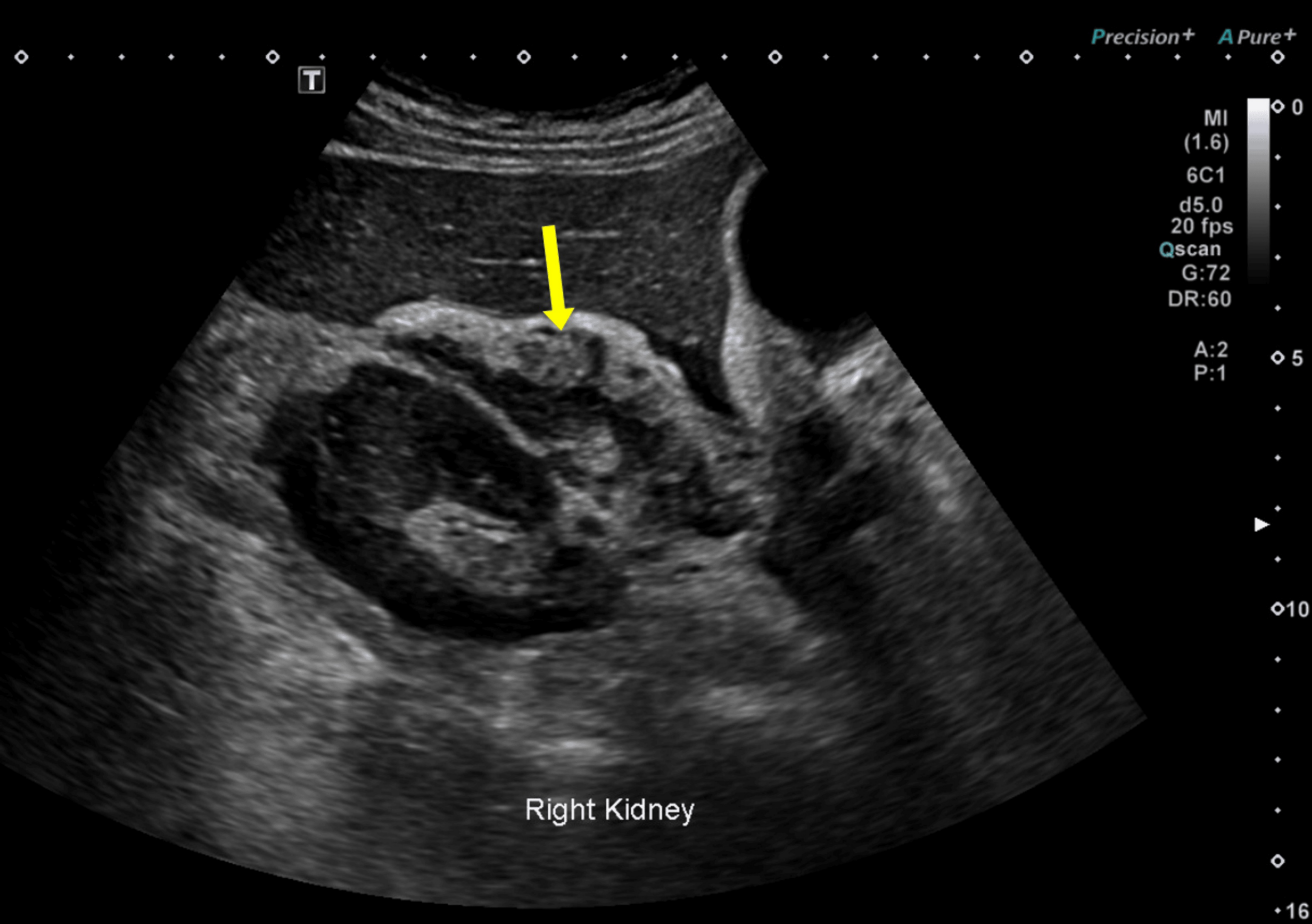
Follow-up ultrasound performed two weeks later demonstrating a heterogeneous suprarenal lesion (yellow arrow).

Two weeks later, the patient returned with recurrent right-sided abdominal pain. A repeat ultrasound demonstrated a sizeable heterogeneous, hypoechoic lesion in the suprarenal and precaval region, which had not been evident on the initial scan (Figure 2). These findings raised suspicion of a collection, abscess, or neoplasm.

**Figure 3:**
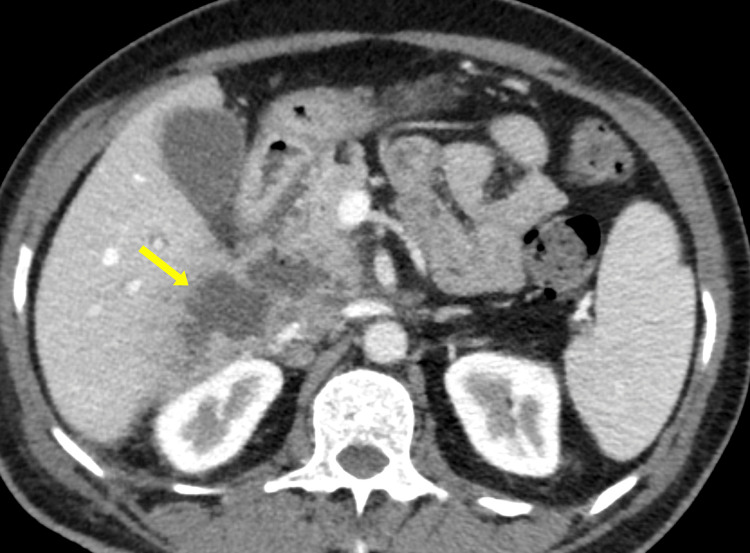
Contrast-enhanced CT scan of the abdomen (axial section) initially interpreted as a right retroperitoneal collection (yellow arrow) due to a sealed duodenal perforation.

A contrast-enhanced computed tomography (CECT) scan of the abdomen was ordered for better characterization. The CECT revealed a large, complex fluid collection in the right anterior pararenal space, closely related to the duodenum, with surrounding fat stranding. The lesion’s borders appeared indistinct, raising concern for a possible sealed perforation or abscess (Figure 3).

**Figure 4:**
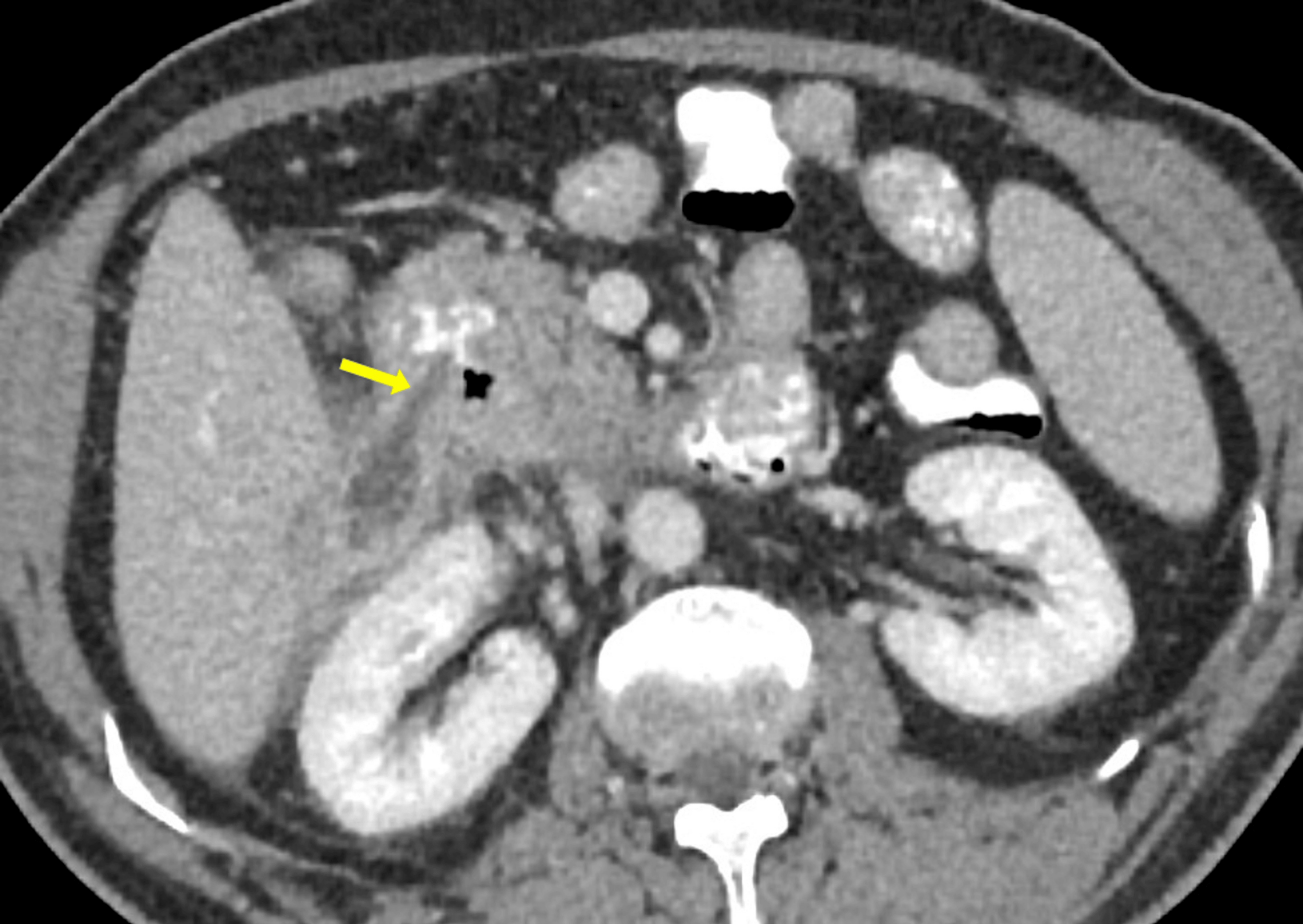
Contrast-enhanced CT scan with intravenous and oral contrast (axial section) showing a narrow tract (yellow arrow) communicating between the lateral wall of the second part of the duodenum and the diverticulum.

Given the suspicion of duodenal pathology, a CT scan with oral contrast was performed prior to percutaneous drainage. Interestingly, the collection did not fill with oral contrast, but features such as mucosal folds within the wall and a narrow communication with the duodenum became apparent upon close inspection. These features suggested a large duodenal diverticulum rather than an abscess or necrotic tumor (Figure 4).

**Figure 5:**
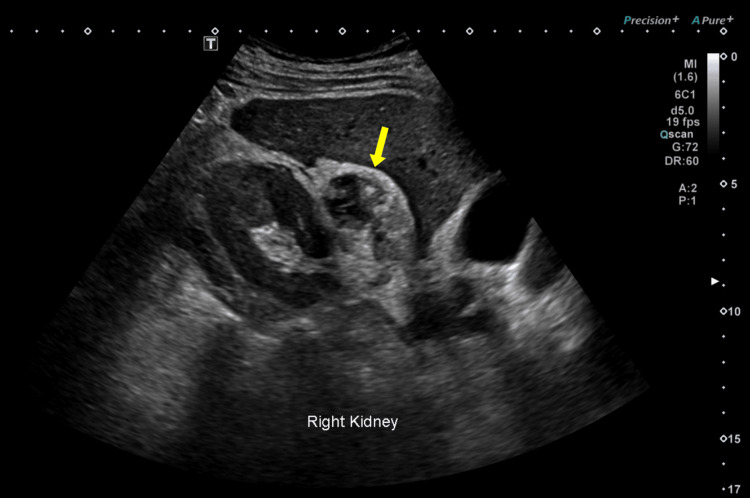
Retrospective ultrasound review revealing bowel wall signature within the suprarenal lesion (yellow arrow), supporting the diagnosis of a duodenal diverticulum.

Review of prior ultrasound images, with attention to subtle features, revealed a bowel wall signature, a thin, echogenic layer consistent with soft tissue layering supporting the presence of a diverticulum (Figure 5). No definitive fistula or contrast passage was observed, likely due to edema and inflammation causing a sealed or narrowed orifice.

Given these findings, the diagnosis of a giant duodenal diverticulum was made. The decision was to avoid invasive drainage and pursue conservative management with intravenous antibiotics. An elective gastroduodenoscopy was planned to confirm the diagnosis and assess for complications; however, the patient was lost to follow-up before the procedure.

## Discussion

Duodenal diverticula are frequently encountered incidental findings, but giant or inflamed diverticula are less common and may present with non-specific symptoms that overlap with other intra-abdominal or retroperitoneal pathologies [3,6]. In such cases, misinterpretation of imaging can lead to an erroneous working diagnosis, as in the patient of this case who was initially thought to have a right retroperitoneal collection secondary to pyelonephritis or a sealed duodenal perforation.

When symptomatic, duodenal diverticula may present with nonspecific clinical features such as abdominal pain, fever, or anorexia [7,8]. Complications are uncommon but may include inflammation, perforation, hemorrhage, intestinal obstruction, and Lemmel’s syndrome, a condition in which a large periampullary diverticulum causes bile duct obstruction and features of obstructive jaundice [3,5,9,10]. Giant duodenal diverticula (>4 cm) are particularly rare and, when inflamed, may mimic other intra-abdominal pathologies such as abscesses, necrotic tumors, or pseudocysts.

Imaging plays a critical role in differentiating diverticula from other conditions. On ultrasound, characteristic findings include bowel wall signatures such as layered echogenicity, peristaltic movement, or wall stratification [11]. In our case, the initial differential diagnosis included pyelonephritis with perinephric involvement, retroperitoneal abscess, and sealed duodenal perforation. Pyelonephritis was initially favored based on clinical presentation and renal ultrasound findings; however, subsequent CT demonstrated a suprarenal lesion closely related to the duodenum, making a renal source less likely and prompting reconsideration of the diagnosis. Thus, the absence of a suprarenal lesion on the initial ultrasound, yet its appearance on the second study, likely reflects the filling and emptying behavior of the diverticulum. On CT, identification of mucosal folds within a fluid collection and a narrow tract of communication with the duodenum are key features; inflammatory changes such as wall thickening and surrounding fat stranding may also be present [12]. In our case, the absence of oral contrast filling was initially misleading, but retrospective review demonstrated duodenal wall communication through a narrow tract. In summary, imaging features favoring duodenal diverticulitis over a retroperitoneal abscess included the presence of internal mucosal folds, identification of a narrow communication with the second part of the duodenum, variable sonographic appearance between examinations, and a bowel wall signature on retrospective ultrasound review. After confirming the diagnosis of a giant duodenal diverticulum, conservative management was preferred because the patient was clinically stable, there was no free perforation, and imaging indicated a contained inflammatory process.

Importantly, if percutaneous drainage had been pursued under the initial misdiagnosis of a retroperitoneal collection, iatrogenic perforation of the diverticulum could have occurred with potentially serious consequences. This case emphasizes the importance of correlating multimodality imaging findings. The identification of mucosal folds on CT prompted re-examination of prior ultrasound images, which revealed a bowel wall signature consistent with a diverticulum [13]. Such retrospective review can be decisive in establishing the correct diagnosis and preventing inappropriate interventions.

## Conclusions

Giant duodenal diverticula are rare but clinically significant entities that may closely resemble retroperitoneal abscesses or cystic lesions. Recognition of characteristic imaging features, such as the presence of mucosal folds and a narrow communication with the duodenal lumen, is crucial for distinguishing these from pathological collections. This case emphasizes that a comprehensive, multimodal imaging approach can prevent unnecessary invasive procedures and guide appropriate conservative management. Radiologists and clinicians should maintain a high index of suspicion for duodenal diverticulitis when evaluating right-sided retroperitoneal lesions with atypical features.
